# Bringing it all together – Gemeinschaftlich aktiv lernen am virtuell geteilten Bildschirm in der Hochschule und digital

**DOI:** 10.1365/s40702-021-00792-2

**Published:** 2021-09-27

**Authors:** Jens Kaufmann, Sayed Hoseini, Pascal Quindeau, Christoph Quix, Sylvia Ruschin

**Affiliations:** 1 Fachbereich Wirtschaftswissenschaften, Hochschule Niederrhein, Mönchengladbach, Deutschland; 2 Fachbereich Elektrotechnik und Informatik, Hochschule Niederrhein, Krefeld, Deutschland; 3 Arbeitsbereich Hochschuldidaktik, Hochschule Niederrhein, Mönchengladbach, Deutschland

**Keywords:** Kollaboration, Digitales Lernen, Data Science, Datenbanksysteme, Pair Programming, Softwareauswahl, Collaboration, Digital Learning, Data Science, Database Systems, Pair Programming, Software Identification

## Abstract

Hochschulen wie Studierende stehen vor der Herausforderung, zukunftsorientierte Fähigkeiten mit teils erkennbar technischem Fokus zu vermitteln bzw. aufzubauen. Dazu gehört neben fachlichen Aspekten auch die Fähigkeit zu (digitaler) Kollaboration. Verstärkt durch die Umwälzungen im Studienbetrieb während der Corona-Pandemie sind dazu geeignete Lehr-Lern-Konzepte gefragt. Dieser Beitrag zeigt die Konstruktion und mögliche Umsetzung solcher Konzepte an Inhalten eines (Wirtschafts‑)Informatik-Studiums auf. Dazu werden zwei Forschungsgegenstände untersucht. Zum einen der Grad an gemeinschaftlicher Aktivität, den Studierende in verschiedenen solcher Lehr-Lern-Szenarien erreichen, zum anderen der Einfluss auf die Bereitschaft von Studierenden zur intensiven Auseinandersetzung mit technischen Studieninhalten. Hierzu werden didaktische Überlegungen im Allgemeinen und an konkreten Veranstaltungen in den Bereichen Datenbanksysteme und Data Science im Besonderen dargestellt. Auf dieser Basis wird gezeigt, welche Eigenschaften eine technische Plattform zur Umsetzung aufweisen muss, und wie eine solche ausgewählt und implementiert werden kann. Die im Rahmen des Anfang 2021 gestarteten Lehrforschungsprojekts IoHubHN bisher gewonnenen Erkenntnisse bilden eine Grundlage für weitergehende Evaluierungen und die Einbettung des Vorgehens in den Ansatz des Scholarship of Teaching and Learning. Die vorgestellten Konzepte und Plattformen sollen dabei prinzipiell auch auf andere Studienfächer übertragbar sein und sind nicht auf rein digital durchgeführte Lehrveranstaltungen beschränkt.

## Abstract (en)

Universities and students both face the challenge to teach, respectively to acquire, future-oriented skills, that partially tend to have a clear technical focus. Among those is the ability to work collaboratively, especially in a digital context. The changes in higher education during the months of the coronavirus pandemic enforce the need to create suitable teaching and learning concepts. This article shows how such concepts and their implementation can be conducted and exemplifies this with two modules from study programs in (business) informatics studies. The discussion addresses two research items. Firstly, this is the amount of collaborative activity that students in these scenarios achieve and secondly it discusses the influence on the students’ willingness to work on technical topics in depth. To show this, it discusses ideas on higher education development in general as well as fitted to specific courses in the fields of database systems and data science. It describes which properties a technical platform must provide if it should be used as an operational basis. Furthermore, it is discussed how such a platform can be chosen and implemented. Insights that have been gathered so far during the first months of the research project IoHubHN serve as a foundation for further evaluation within the framework of Scholarship of Teaching and Learning (SoTL). Concepts and platforms in this context should be usable for other fields of study as well and are not limited to pure virtual classes.

## Einleitung

Deutsche Hochschulen sind aufgefordert, digitale Schlüssel-Qualifikationen von Studierenden zu stärken. Eine 2018 veröffentlichte Studie von Stifterverband und McKinsey zu Future Skills zeigt nicht nur, dass bis 2023 ein erheblicher Mehrbedarf an Personen mit technologischen Fähigkeiten (z. B. Datenanalyse) bestehen wird, sondern verweist auf die Notwendigkeit, Hochschulabsolvierende für den Arbeitsmarkt mit digitalen Grundfähigkeiten auszustatten. Zu diesen gehört Kollaboration – auch über Distanzen und die Grenzen der eigenen Disziplin hinaus (Kirchherr et al. 2018).

Um Lehre an Hochschulen so zu gestalten, dass digitale Fähigkeiten erworben, Kollaboration gefördert und damit inhärent auch die Kompetenz zu kollaborativer Zusammenarbeit erworben wird, können Software-Lösungen zum Einsatz kommen, die geeignete Lehrkonzepte unterstützen. Dies wird noch relevanter unter dem Eindruck der sogenannten Corona-Semester. Befragungen von Studierenden zeigen, dass insbesondere der persönliche Austausch mit anderen Studierenden vermisst wird (Boros et al. 2020; Traus et al. 2020), wobei die Gesamtsituation einzelne Studierendengruppen, z. B. Studierende mit Kind, besonders belastet (Zimmer et al. 2021). Gleichzeitig hat dieser externe Effekt die Digitalisierung der Lehre an den Hochschulen angetrieben, neue Lehrformate bestärkt und Digitalisierungsstrategien hervorgebracht oder deren Umsetzung beschleunigt (Goertz und Hense 2021).

Dieser Beitrag zeigt dazu passende, existierende und erprobte Lehr-Lern-Konzepte auf. Es wird dargestellt, wie diese an ausgewählten Kerninhalten eines Wirtschaftsinformatik-Studiums angewendet werden können und welche Potenziale existieren, dies auf weitere, auch nicht-technische Fächer zu übertragen. Konkret werden im Rahmen des Lehrforschungs-Projektes IoHubHN an der Hochschule Niederrhein zwei Lehrveranstaltungen der Wirtschaftsinformatik zu Datenbanksystemen und Data Science herausgegriffen, an denen didaktische wie technische Konzepte erarbeitet, umgesetzt und evaluiert werden.

Ziel ist es, die Kollaboration der Studierenden durch den Einsatz einer webbasierten Software zu unterstützen. Die Software ermöglicht das gleichzeitige Bearbeiten von Aufgaben und Lösungen und stellt die Aktionen aller Beteiligten nahezu in Echtzeit dar. In der betrieblichen Praxis wie auch in der Informatik-Ausbildung wird die Technik des Pair Programming erfolgreich eingesetzt (Reinhardt 2007). Methoden dieses Konzepts werden hier aufgegriffen und auf den fachlichen Kontext der Lehrveranstaltungen angepasst und angewendet. Im Rahmen der Bemühungen ergeben sich so zwei Forschungsgegenstände.

1. Untersucht wird, welchen Grad an gemeinschaftlicher Aktivität Studierende in verschiedenen Lehr‑/Lernszenarien erreichen und welchen Effekt Studierende heterogener Leistungsniveaus dem Einsatz des Lehrkonzepts zuschreiben.
2. Untersucht wird, inwiefern die kollaborative Nutzung der Software die Bereitschaft der Studierenden steigert, sich intensiv mit (technischen) Studieninhalten zu beschäftigen.

Ein essenzieller Bestandteil der Aktivitäten ist zudem die Identifizierung der richtigen technischen Plattform, insbesondere einer geeignet einsetzbaren Software und deren angemessener Konfiguration sowie praktischer Einbindung in den Lehrbetrieb und in existierende IT-Systeme. Aus der vorgestellten Motivation, den Forschungsfragen und den (geplanten) Aktivitäten ergeben sich mehrere Beiträge dieses Artikels zum wissenschaftlichen Diskurs in folgender Struktur:

Abschn. 2 stellt das kompetenztheoretische Fundament kollaborativer Ansätze vor und liefert einen Überblick zu Forschungserkenntnissen in diesem Bereich. Im Anschluss werden hier am Beispiel der konkreten Veranstaltungen mögliche Ansätze für fachdidaktische Konzeptionierungen im gegebenen Kontext gezeigt.

Abschn. 3 bietet eine Übersicht möglicher technischer Umsetzungen, stellt Vor- und Nachteile unterschiedlicher Software-Lösungen dar und erläutert den Auswahl- und Implementierungsprozess unter Berücksichtigung der vorgestellten Lehr-Lern-Szenarien.

Im vierten Abschnitt wird das methodische Design zur Überprüfung der Lehrforschungsfragen vorgestellt und in das theoretische Konstrukt des Scholarship of Teaching and Learning (SoTL) eingebettet.

Alle vorgestellten Überlegungen werden derzeit an der Hochschule Niederrhein im Rahmen des Projektes IoHubHN entwickelt und durchgeführt. IoHubHN wird im Rahmen der landesweit geförderten „Fellowships für Innovationen in der digitalen Hochschullehre“ finanziert und setzt die vorgestellten Konzepte in den Jahren 2021 und 2022 als Pilot an der Hochschule um. Abschn. 5 führt die im Projekt bisher gesammelten Erkenntnisse zusammen und gibt einen Ausblick auf die noch folgenden Aktivitäten und zukünftig erwarteten Ergebnisse in Bezug auf die Forschungsgegenstände.

## Fachdidaktische Konzeptionierung

Hochschulen haben einen gesellschaftlichen Bildungsauftrag (Hochschulrahmengesetz 2019). Zu vermittelnde Kompetenzen wie der Umgang mit digitalen Technologien – etwa digitale Wissensgenerierung, digitales Problemlösen – und kollaborative Zusammenarbeit sind zentrale Voraussetzungen für gesellschaftliche Teilhabe und wirtschaftlichen Erfolg (Kirchherr et al. 2018).

### Kompetenzorientierung und didaktisches Design

In modernen Gesellschaften sind Wissen und Wissenschaft von herausragender Bedeutung für die Weiterentwicklung der Gesellschaft. Damit einher gehen einerseits eine Spezialisierung in den Wissenschaftsbereichen, andererseits eine stark abnehmende Halbwertzeit von Wissen. Das wirkt sich auf die Prozesse der Vermittlung von Wissen und den Erwerb von Kompetenzen insofern aus, als dass in das Zentrum von Lehr-Lern-Prozessen stärker die Fähigkeit rückt, aus der Menge zugänglicher Informationen die für bestimmte Sachverhalte relevanten auszuwählen und (für die eigenen Bedarfe) auswerten zu können. Entsprechend ist das kanonische Wissen um prozedurale und metakognitive Strategien der Wissensaneignung zu ergänzen. Hier kommt das Postulat der Kompetenzorientierung zum Tragen und wird verknüpft mit dem Auftrag einer grundsätzlichen Beschäftigungsbefähigung (*employability*) von Absolventinnen und Absolventen (vgl. z. B. Wissenschaftsrat (2000), insbesondere S. 21f.).

Im didaktischen Verständnis von Hochschullehre lassen sich zwei grundsätzliche Deutungen des Begriffs der Kompetenzorientierung identifizieren, die unterschiedliche Implikationen für die Gestaltung von Lehre und des studentischen Lernens haben. Insbesondere an Hochschulen für angewandte Wissenschaften wird häufig aus dem Umfang dargelegter Schlüsselqualifikationen auf den Grad der Kompetenzorientierung von Lehr- und Lernangeboten (und der Beschäftigungsbefähigung von Studierenden) geschlossen. Dieser Kompetenzbegriff beruht auf einem Berufsbildungsverständnis, das seine Wurzeln im 20. Jahrhundert hat und das fachliche Kompetenzen, Methodenkompetenzen, soziale sowie personale Kompetenzen voneinander zu unterscheiden sucht. Im Unterschied dazu geht der wissenschaftlich-akademische Kompetenzbegriff, wie er von Schaper et al. (2012) im Rahmen eines Fachgutachtens für die Hochschulrektorenkonferenz entwickelt wurde, davon aus, dass es zu „integrierende[r] Bündel von komplexem Wissen, Fertigkeiten, Fähigkeiten, motivationalen Orientierungen und (Wert‑)Haltungen“ bedarf, um „in Anforderungsbereichen, die durch hohe Komplexität, Neuartigkeit, Unbestimmtheit und hohe Ansprüche an die Lösungsqualität gekennzeichnet sind, angemessen, verantwortlich und erfolgreich“ handeln zu können (Schaper et al. 2012, S. 29). Kompetenzorientierung reicht nach diesem Verständnis über den Erwerb von Wissen weit hinaus. Kompetenzorientiertes Lehren, Lernen und Prüfen nimmt den handelnden Umgang mit Wissen und die Bewältigung komplexer Anforderungen und Aufgabenstellungen in den Blick.

Dieser akademische Kompetenzbegriff liegt dem didaktischen Design der beiden Lehrveranstaltungen „Datenbanken“ sowie „Data Science“ zugrunde, die im Rahmen des Projektes IoHubHN weiterentwickelt werden. Kompetenzorientiertes Lehren und Lernen erhöht die Anforderungen sowohl an die Lehrenden als auch an die Studierenden. Während von jenen gefordert ist, dass sie entsprechende Lernangebote machen, sind diese gehalten, sich aktiv in das Lerngeschehen einzubringen und ihren eigenen Lernprozess verantwortlich mitzugestalten. In den Corona-Semestern der Jahre 2020/21 hat sich eine Diskrepanz aufgetan zwischen der erwarteten sozialen Interaktion und der tatsächlichen Interaktion zwischen den Studierenden. Interaktion ist allerdings zentrale Voraussetzung für kompetenzorientiertes Lehren und Lernen.

### Kollaborative Zusammenarbeit: Pair Programming und das Erzeugen von Gruppendynamik

Vor allem unter Personen, die Software entwickeln, hat sich Kollaboration als essenzieller Bestandteil der täglichen Arbeit etabliert (Goel und Kathuria 2010). So nutzen Softwareunternehmen agile Methoden des *EXtreme Programming*, zu denen auch das Pair Programming gehört. Studien belegen den positiven Effekt auf die Lerneffektivität von Studierenden durch eine Steigerung der Lösungsqualität bei gleichzeitig reduziertem Zeitaufwand (Hannay et al. 2009).

Als ausschlaggebend für den Erfolg von Pair Programming in der Lehre hat sich die Interaktion zwischen Studierenden (einer Arbeitsgruppe) erwiesen – sofern sie eine bestimmte Gruppengröße nicht überschreiten (Saqr et al. 2019). Zu den positiven Effekten einer hierbei eingesetzten kollaborativen Lernumgebung gehören: *Ergänzendes Wissen, Fehlerkorrektur* und *Reflexion von Wissen* als kognitive Vorteile und *Erhöhtes Engagement* sowie das *Einsehen mehrerer Perspektiven* als soziale Vorteile (Nokes-Malach et al. 2015). Je größer die Gruppe, desto höher ist allerdings das Risiko des sogenannten Trittbrettfahrens (*Social Loafing*). Eine Ursache hierfür mag sein, dass die Zusammenarbeit von Studierenden zeitlich begrenzt ist. Daher wird abhängig von der Komplexität der Aufgabe üblicherweise eine kleine Gruppengröße von drei Studierenden gewählt (Lohman und Finkelstein 2000; Tu und McIsaac 2002) – insbesondere, wenn es um eine kollaborative Echtzeitumgebung geht, wie sie hier angestrebt wird. Kollaboration in Echtzeit bedeutet in diesem Zusammenhang, dass mehrere Benutzer:innen parallel an einem gemeinsamen Dokument (z. B. Quellcode oder Textdokument) arbeiten können und die Änderungen der einzelnen Benutzer:innen mit sehr geringer Verzögerung (weniger als eine Sekunde) von der Software zusammengeführt werden.

Für den Erfolg einer kollaborativen Zusammenarbeit ist zudem die Gruppenzusammensetzung relevant. Die Studierendengruppen können dabei entweder durch eine Zufallsauswahl oder durch Selbstauswahl zusammengestellt werden. Chapman et al. (2006) zeigen, dass Studierende, die ihre Gruppe selbstständig wählen, besser miteinander kommunizieren, eine verbesserte Fähigkeit zur Konfliktlösung ausbilden und mehr Freude an der Arbeit innerhalb der Gruppe entwickeln. Demgegenüber zeigen Studierende, die zufällig in Gruppen eingeteilt werden, eine bessere Zeiteffizienz und Arbeitsorientierung. Die kommunikativen Vorteile der Selbstauswahl begünstigen hierbei den Kompetenzerwerb.

Der Erfolg des Pair Programming motiviert dazu, den dort verwendeten kollaborativen Ansatz in den vorliegenden Kontext zu transferieren. Der Ansatz wird daher zur didaktischen Konzeption und Begründung der kollaborativen Lehr-Lern-Szenarien verwendet, die den beiden Lehrveranstaltungskonzepten zugrunde liegen.

### Lehr- und Lernszenarien im gegebenen Kontext

Die Implementierung einer kollaborativen Lehr- und Lernumgebung erfolgt im hier beschriebenen Projekt konkret in zwei Lehrveranstaltungen der (Wirtschafts‑)Informatik. Die Veranstaltung *Datenbanken* findet regulär im dritten Semester des Bachelor-Studiengangs Wirtschaftsinformatik statt, die Veranstaltung *Data Science* im fünften Semester des Bachelor-Studiengangs Informatik. Beide Veranstaltungen wurden wegen ihres eindeutig (wirtschafts-)informatischen und somit technischen Kontextes ausgewählt. Zum einen sind hier technische Kompetenzen essenzieller Bestandteil des Curriculums, zum anderen kann davon ausgegangen werden, dass Studierende dieser Fachrichtung dem Erwerb von Data Literacy, Kollaboration und digitalem Lernen aufgeschlossener gegenüberstehen als andere Studierendengruppen (Senkbeil et al. 2019), sodass sich die Frage nach einer bereitwilligen intensiven Beschäftigung mit der fachlichen Thematik ohne eventuelle verzerrende Effekte genereller Ablehnung des Lehr-Lern-Szenarios beantworten lässt.

In der Veranstaltung *Datenbanken* erlernen die Studierenden neben theoretischen Grundlagen auch praktische Kenntnisse. Explizites Ziel ist es, dass Studierende komplexe Datenbankabfragen, Datendefinitionen und -änderungen mit Hilfe der Datenbankabfragesprache SQL programmieren können. Dazu wird in der Lehrveranstaltung eine Online-Umgebung zur Verfügung gestellt. Studierende können sich per Selbstauswahl einer Studiengruppe zuordnen. Die Studierendengruppen haben die Möglichkeit, die Online-Umgebung für die Übungen und außerhalb der Veranstaltungszeiten auch für die Arbeit an selbstgewählten Fragestellungen zu nutzen.

Zentral für den fachlichen Kompetenzerwerb und den Erwerb von Fähigkeiten kollaborativer Zusammenarbeit ist eine gut und angemessen komplex konzipierte Aufgabenstellung. Übungsaufgaben sehen daher beispielsweise vor, dass Datenbankstrukturen mit mehreren Tabellen erzeugt werden müssen, die dann über SQL-Befehle mit fiktiven Daten befüllt werden, sodass sich Schemata ergeben, die Selektion, Projektion und Verbundanfragen (Join) in unterschiedlicher Ausprägung unterstützen. Studierende erhalten dann eine Fragestellung mit Beispielausgabe und müssen einen allgemein gültigen Abfrage-Befehl entwickeln, der die Fragestellung auch nach Veränderung der Datenbankinhalte korrekt beantwortet. Durch die Hinzunahme von mehreren Tabellen, Aggregatfunktionen, Unterabfragen und weiteren komplexeren Abfragebestandteilen kann der Schwierigkeitsgrad sukzessive erhöht werden.

**Abb. 1:**
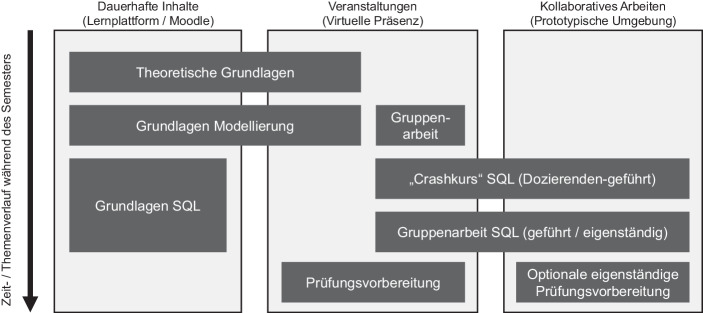
Schematisierte, verkürzte Darstellung des Zusammenhangs von Lernplattform, virtuellen Präsenzveranstaltungen und Einsatz der kollaborativen Plattform im Kurs „Datenbanken“

In Live-Sessions werden von den Lehrenden derartige Aufgaben bereitgestellt, die in Gruppen unter festen zeitlichen Vorgaben bearbeitet werden. Ziel ist es, dass die Studierenden kollaborativ eine Serie von Aufgaben bearbeiten und den kontinuierlich steigenden Schwierigkeitsgrad gemeinsam bewältigen. Lehrende unterstützen den Prozess bei Bedarf. Eine Diskussion in der Gesamtgruppe und das Einnehmen von Expertenrollen durch fortgeschrittene Studierende schließen die Live-Veranstaltungen ab. Abb. 1 stellt den Zusammenhang unterschiedlicher Lernangebote in der Veranstaltung schematisch dar.

Ein vergleichbares Szenario trifft auf die *Data-Science*-Veranstaltung zu, in der verschiedene Methoden zur Datenaufbereitung und -analyse, aber auch die mathematischen Grundlagen dieser Methoden vermittelt werden. Im Vergleich zur Datenbank-Veranstaltung sind die Aufgabenstellungen umfangreicher und erfordern weitergehende Kenntnisse, da es sich um eine Wahlpflichtveranstaltung für Studierende im fünften Semester des Studiengangs Informatik handelt. Studierende sollen (kurze) Python-Programme erstellen, die Daten für gegebene Fallstudien aufbereiten und durch Machine-Learning-Methoden analysieren. Das notwendige Grundlagenwissen wird den Studierenden vorher in Vorlesungen in Präsenz bzw. über interaktive Lerneinheiten (z. B. *H5P*^1^) vermittelt. In Live-Sessions werden die Studierenden auf die Übungsaufgaben durch entsprechende Beispiele vorbereitet. Die Programmieraufgaben sind offen gestellt und ermöglichen verschiedene Lösungswege, erfordern aber auch komplementäre Kompetenzen aus den verschiedenen Data-Science-Bereichen (u. a. Datenaufbereitung, Analyse, Statistik, Visualisierung). So ergibt sich der Kollaborationsbedarf unmittelbar aus den verschiedenen Kompetenzen, die ein Data Scientist vereinen sollte und die in der Veranstaltung erworben werden sollen. Der Zeitraum zur Bearbeitung der Aufgaben ist den Studierenden freigestellt, aufgrund der umfangreicheren Aufgabenstellung findet dies aber üblicherweise nicht in den Live-Sessions statt. Nach einer Bearbeitungszeit von ein bis zwei Wochen sollen die Studierenden ihre Lösungen abgeben, die dann bewertet und in einer weiteren Live-Session besprochen werden.

Der Einsatz des Live-Session-Konzeptes ist dabei nicht nur unter Bedingungen digitaler Lehrveranstaltungen nutzbar, sondern unterstützt auch Präsenzveranstaltungen. Dies ist insbesondere der Fall, wenn die Kursgröße (z. B. deutlich zweistellig) oder die zur Verfügung stehende Raumsituation (z. B. Festbestuhlung) keine kollaborative Arbeit in Kleingruppen unterstützt.

## Auswahl und Implementierung einer geeigneten technischen Umgebung

Zur Bewältigung der oben skizzierten Herausforderungen muss eine entsprechende kollaborative Lehr-Lern-Umgebung geschaffen werden. Hierfür sollen zunächst die Anforderungen an eine solche Umgebung aufgezeigt werden. Diese können für die Auswahl einer Softwarelösung herangezogen werden, wenn bereits vorhandene Kollaborationsplattformen anhand verschiedener Kriterien miteinander verglichen werden.

### Abgeleitete Anforderungen aus dem fachdidaktischen Konzept

Kollaboration setzt eine Vielzahl von technischen Anforderungen voraus, damit eine effektive Zusammenarbeit überhaupt ermöglicht werden kann. Am Beispiel von Data Science führen Wang et al. (2019) dazu eine umfangreiche Untersuchung durch. Data Scientists arbeiten domänenübergreifend in interdisziplinären Teams zusammen, um komplexe Sachverhalte und Datenbestände explorieren, visualisieren und modellieren zu können, was sowohl synchrone als auch asynchrone Kollaboration erfordert.

Zu den größten Herausforderungen einer synchronen Echtzeit-Kollaboration gehören *Interference* und *Lack of Awareness*. *Interference* bezeichnet Änderungen (z. B. von Variablennamen) ohne dies ausreichend mit den Kollaborationsparteien kommuniziert zu haben. Um das Auftreten solcher Code-Interferenzen zu vermeiden, haben laut der Studie Teilnehmende Code-Segmente für sich beansprucht und Änderungen verbal unterbunden. Hieraus lässt sich als mögliche Funktionalität das Sperren von Code-Segmenten ableiten. *Lack of Awareness* umfasst demgegenüber das fehlende Bewusstsein für die Aktivitäten aller Gruppenmitglieder. Ein Nutzer-Cursor, der die aktuelle Position aller Nutzer:innen im Code anzeigt, könnte dieses Bewusstsein verbessern, wurde aber weiterhin als unzureichend empfunden (Wang et al. 2019). Eine Alternative könnte eine Änderungshistorie sein, die von allen Gruppenmitgliedern eingesehen werden kann. Somit wird eine effiziente Nachvollziehbarkeit von vergangenen Aktivitäten (Historie) und aktuellen Aktivitäten (Nutzer-Cursor) kombiniert und erhöht das Bewusstsein für die Bearbeitung der anderen Kollaborationsparteien.

Unabhängig von den oben genannten Herausforderungen erweist sich die Möglichkeit eines schnellen Austauschs als positiv für eine erfolgreiche Kollaboration. Der Wechsel zwischen Arbeits- und Kommunikationssoftware wird als hinderlich empfunden (Wang et al. 2019). Wünschenswert wäre daher eine Plattform, die sowohl Programmier-Funktionalitäten als auch Kommunikations-Funktionalitäten (Chat, Voice-Call) verbindet. Weitere Merkmale umfassen eine eingebundene Dateiverwaltung sowie die direkte Integration einer Versionsverwaltung (z. B. *GitLab*). Dies würde die Nutzung externer Software-Komponenten minimieren, sodass alle notwendigen Tätigkeiten direkt in der Kollaborationsumgebung durchgeführt werden können.

Damit einher geht die Anforderung der Integration der Kollaborationsplattform in eine Lernplattform (z. B. *Moodle*). Lerninhalte wie Aufgabenstellungen, Beispiele und Lösungen sollten ohne manuellen Datei-Up- oder -Download direkt zwischen den verschiedenen Systemen übertragen werden können. Das bedeutet auch, dass die Kollaborations- und Programmier-Plattform Single-Sign-On (SSO – ein Benutzeraccount für die verschiedenen Plattformen) und eine vollständig web-basierte Benutzeroberfläche bereitstellen muss, die neben einem Web-Browser keine zusätzliche Software bei den Studierenden erfordert. Auf Server-Seite müssen daher entsprechende Vorkehrungen für Datenschutz und Mehrbenutzerbetrieb getroffen werden. Dazu gehören z. B. ausreichende System-Ressourcen, geringe Antwortzeiten, Skalierbarkeit, Isolation der verschiedenen Umgebungen der einzelnen Gruppen und Wiederherstellbarkeit bei System-Fehlern.

### Positionierung und Gegenüberstellung verschiedener virtueller Kollaborationsumgebungen

Im Folgenden wird ein Vergleich verschiedener virtueller Kollaborationsumgebungen vorgenommen. Für den Einsatz in den beschriebenen Lehrveranstaltungen sollte eine Plattform die Kollaboration in Echtzeit, Data-Science-Programmiersprachen wie *Python, SQL* oder *R*, integrierte Kommunikationstools (Chat/Video), SSO und Skalierbarkeit unterstützen. Da Studierende in einer Lehrveranstaltung nicht gezwungen werden können, ihre personenbezogenen Daten einer (ausländischen) Cloud-Plattform zu übergeben, sind aus Datenschutzgründen Open-Source-Lösungen, die einen Einsatz „On-Premise“ erlauben, gegenüber Cloud-Lösungen zu bevorzugen. Cloud-Lösungen wurden im Vergleich dennoch betrachtet, da ein deutlich größerer Funktionsumfang als bei Open-Source-Systemen eine aufwändige datenschutzrechtliche Prüfung und entsprechende technische Lösung rechtfertigen würde (wie es z. B. viele Hochschulen bei Video-Konferenz-Plattformen gemacht haben). Entwicklungsumgebungen mit Echtzeit-Kollaboration, die eine lokale Software-Installation auf Seiten der Nutzer:innen erforderlich machen, wurden nicht betrachtet (z. B. *CodeWithMe*). Informatik-Studierende hätten mit einer entsprechenden Lösung mit lokaler Installation zwar wenig Probleme, da die Plattform später aber auch in nicht-technischen Studiengängen eingesetzt werden soll, werden web-basierte Plattformen vorgezogen.

Eine bekannte Programmier-Umgebung für Data Science ist *Jupyter*, womit verschiedene Data-Science-Aufgaben in einer webbasierten Oberfläche umgesetzt werden können. In sogenannten *Computational Notebooks* (Wang et al. 2019) können neben Programmfragmenten auch erklärende Textbausteine oder Videos eingefügt werden. *Jupyter* ist aufgrund der langjährigen Entwicklung und großen Community eine ausgereifte Plattform, bietet aber in der Standardimplementierung keine Echtzeit-Kollaboration. Das Basissystem des *Jupyter*-Projekts ist das *Jupyter Notebook*, mittlerweile gibt es mit *JupyterHub* auch eine skalierbare Lösung. *JupyterLab* ist eine weitere Variante, die seit Mitte 2021 auch Echtzeit-Kollaboration unterstützt. Diese Funktionalität befindet sich dort aber aktuell noch weiter in Entwicklung. Die Angebote der Firmen DataBricks und DeepNote haben sehr umfassende, dem Jupyter Notebook ähnliche Funktionen und bieten auch Echtzeit-Kollaboration. Selbiges gilt auch für *Google Colab*, welches kurzzeitig ebenfalls Echtzeit-Kollaboration ähnlich zu *Google Docs* ermöglichte, die jedoch wieder entfernt wurde. Diese Angebote sind zwar proprietäre Cloud-Lösungen, sie bieten aber einen großen Funktionsumfang an, der von Open-Source-Lösungen ebenfalls erreicht werden sollte. *Apache Zeppelin* bietet eine weitreichende Unterstützung für verschiedene Programmiersprachen, Datenbank- und Big-Data-Systeme (insbesondere *Apache Spark*). *Zeppelin* implementiert sowohl Echtzeit-Kollaboration als auch die Möglichkeit, verschiedene Programmiersprachen innerhalb desselben Notebooks auszuführen. *CoCalc* baut auf *Jupyter Notebooks* auf und erweitert diese um Echtzeit-Kollaboration, einen Nutzer-Cursor, Daten‑, Benutzer- und Kursmanagement, einen Chatroom und das Videokonferenzwerkzeug *Jitsi*. Mittels *CoCalc* können nicht nur *Computational Notebooks*, die eine bestimmte Programmiersprache bereitstellen, kollaborativ bearbeitet werden, sondern auch die Linux-Kommandozeile, ein *LaTeX*-Editor und in der Vollversion zudem eine Reihe von graphischen Oberflächen.

### Technische Einbindung und Umsetzung

Die Kollaborationsumgebung soll als webbasierte Plattform bereitgestellt werden und für Lernende und Lehrende über einen Web-Browser bedienbar sein. Die Bereitstellung von Web-Anwendungen über Virtualisierungsumgebungen wie z. B. *Docker* oder *Kubernetes* ist heute gängige Praxis, um eine Anwendung skalieren und auf neue Server-Systeme einfach übertragen zu können. Im Projekt IoHubHN wird derzeit ein Kubernetes-Cluster eingesetzt, um *Zeppelin* und *CoCalc* in ersten Lehrveranstaltungen zu testen. Dadurch wird auch die notwendige Isolation zwischen verschiedenen Benutzergruppen erreicht, da jedes *Zeppelin*-Notebook bzw. *CoCalc*-Projekt in einem separaten Container läuft. Des Weiteren können so für verschiedene Benutzergruppen in den verschiedenen Lehrveranstaltungen unterschiedliche Software-Pakete zur Verfügung gestellt werden, ohne dass Konflikte zwischen Software-Paketen bzw. -Versionen zu erwarten sind. Schließlich muss bei einem System-Absturz nur ein einzelner Container und nicht das komplette Server-System wiederhergestellt werden. Weiterhin wird eine Web-Applikation entwickelt, die vor allem das Benutzermanagement über das Identity-Management-System der Hochschule organisieren soll, um es Nutzer:innen zu ermöglichen, sich mit ihrer Hochschulkennung anzumelden (SSO). Der erste Testbetrieb im Sommersemester 2021 hat ergeben, dass Zeppelin zwar sehr gut für eine Veranstaltung zu Big-Data-Technologien geeignet ist, da viele verschiedene Technologien auch innerhalb eines Notebooks genutzt werden können, bezüglich Kollaboration und Kursmanagement bietet CoCalc aber geeignetere Lösungen. Im Wintersemester 2021/22 wird daher CoCalc in den Lehrveranstaltungen *Datenbanken* und *Data Science* eingesetzt.

## Eingliederung des Vorhabens in den Kontext Forschung zum Lehren: Scholarship of Teaching and Learning

Mit Hilfe des Ansatzes des Scholarship of Teaching and Learning (SoTL) wird eine evidenzbasierte Reflexion durch einen explizit forschungsgeleiteten Blick auf die eigene Lehre als Format der Lehrentwicklung etabliert (Huber 2011). Dies dient auch der Erprobung neuer und innovativer Formate und Methoden. Die ad-hoc-Umstellung auf digitale Lehre infolge der Corona-Pandemie wurde an der Hochschule Niederrhein im Frühjahr 2020 zum Anlass genommen, früher als geplant die Beforschung der eigenen Lehre als ein strategisches Handlungsfeld der Lehrentwicklung einzuführen (Eßer-Lüghausen et al. 2021).

Ein SOTL-Vorhaben befasst sich aus wissenschaftlicher Perspektive mit der eigenen Lehre und/oder dem Lernen der Studierenden. Zielsetzung ist es (a) die Ziele, Mittel, Bedingungen und Wirkungen des eigenen Lehrens und/oder des studentischen Lernens hypothesengeleitet und systematisch zu beobachten, um (b) auf dieser Grundlage und vor dem Hintergrund des einschlägigen Forschungsstandes zur Fragestellung die eigene Lehre evidenzbasiert weiterzuentwickeln und (c) die Erkenntnisse öffentlich und damit dem Erfahrungsaustausch und einer breiten Diskussion zugänglich zu machen. Mit dem SoTL-Ansatz werden die subjektiven Theorien und impliziten Annahmen über das Lehren und Lernen sichtbar und damit einer Überprüfung zugänglich gemacht.

Um zu überprüfen, inwieweit sich die Einführung einer Plattform für Online-Kollaboration tatsächlich auf die eingangs formulierten Forschungsgegenstände auswirkt, bedarf es einer geeigneten Evaluierung. Erhoben werden sollen der Grad der (gemeinschaftlichen) Aktivität und inwieweit sich die Bereitschaft steigern lässt, sich intensiv mit (technischen) Studieninhalten zu beschäftigen. Die Studierenden werden als Expert:innen des eigenen Lernens unmittelbar einbezogen. Dies ist insbesondere aufgrund der fehlenden Vergleichbarkeit der gegenwärtigen digitalen Lehrveranstaltungen mit vorhergehenden, in Präsenz durchgeführten Lehrveranstaltungen notwendig. Studentischer Lernerfolg ist multifaktoriell bedingt und nicht alle Aspekte können durch Lehr-Lern-Interventionen adressiert oder kontrolliert werden. Die daher komplizierte Erfassung relevanter Messfaktoren des Erfolgs eines Lehr-Lern-Konzeptes ist zudem erschwert durch die veränderten Rahmenbedingungen während der Corona-Pandemie: Lehrende nehmen eine Abnahme der aktiven Beteiligung von Studierenden im digitalen Lehr- und Lernumfeld wahr (Kreulich et al. 2020). Zugleich wurden kurzfristig neue, digitale und für Lehrende und Studierende in Teilen ungewohnte Prüfungsformate eingeführt. Aus diesem Grund werden Studierende im beschriebenen Vorhaben zu mehreren Zeitpunkten mithilfe eines standardisierten Verfahrens schriftlich befragt. Um die zwei latenten Konzepte zu messen, werden verschiedene Items konzipiert, die zu Fragebatterien zusammengefasst werden. So lassen sich für jede erhobene Größe (aggregierte) Testwerte bestimmen. Anschließend kann aufgrund der mehrfachen Befragung eine Trendanalyse durchgeführt werden. Maßgeblich im Sinne der diskutierten Forschungsgegenstände sind dabei Erfassung und (Selbst‑)Bewertung der inneren Haltung der Studierenden (z. B. die Bereitschaft zu Gruppenarbeit). Die Befragung wird im Wintersemester 2021/22 in den im Abschn. 2.3 beschriebenen Lehrveranstaltungen durchgeführt.

Um einen Vorher-Nachher-Vergleich zu ermöglichen, haben Studierende im Rahmen eines einstündigen Workshops über Unterschiede in der Zusammenarbeit vor und während der Corona-Pandemie reflektiert und Erwartungen an eine webbasierte Kollaborations-Plattform formuliert. Der Workshop wurde im Rahmen einer Masterveranstaltung im Studiengang Informatik durchgeführt. Insgesamt haben sich sechs Studierende dazu bereit erklärt, daran teilzunehmen. Zunächst haben die Studierenden die oben genannten Aspekte in 2er-Gruppen diskutiert. Nach jeder Diskussion wurden die Ergebnisse daraufhin im Plenum vorgetragen und schließlich zusammengeführt. Dabei haben Studierende darauf aufmerksam gemacht, dass sie während der Präsenzveranstaltungen Aufgaben gemeinschaftlich am gleichen Bildschirm bearbeiten konnten, was während der Corona-Pandemie aufgrund des ausschließlich digitalen Lehrformates nicht möglich war. Stattdessen mussten sie auf eine Vielzahl von Kommunikations- und Dateiverwaltungstools zurückgreifen, um effektiv zusammenarbeiten zu können, was durch die Einführung einer kollaborativen Online-Plattform im digitalen Raum vereinfacht werden kann. Weiterhin zeigten die Gespräche mit den Studierenden, dass die Gruppenarbeit bisher vorwiegend nur kooperativ (ca. 70 % der Zeit nach eigener Einschätzung) und in Einzelarbeit stattfindet. Nur die Ergebnisbesprechung und die Bearbeitung besonders komplexer Aufgaben führte zu echter Kollaboration.

Im Rahmen des Workshops wurden die Studierenden zudem gebeten, Eigenschaften einer für sie idealen Plattform zur kollaborativen Zusammenarbeit zu beschreiben. Weiterhin bekamen die Studierenden zwei Stimmen, die sie auf die zuvor genannten Eigenschaften verteilen konnten. So wurden die wichtigsten Aspekte hervorgehoben. Allgemein wurde eine stabil laufende Plattform gefordert. Im Bereich der anwendbaren Funktionalitäten haben sich die meisten Studierenden für einen Nutzer-Cursor ausgesprochen, sodass stets ersichtlich ist, wer gerade woran arbeitet. Weitere als hilfreich identifizierte Funktionalitäten umfassen ein Kommentar- und Annotationswerkzeug und eine Möglichkeit der Nachvollziehbarkeit von Änderungen durch andere Gruppenteilnehmende. Das Feedback der Studierenden beschränkt sich damit nicht nur auf die Bewertung vordefinierter Software-Lösungen, sondern wird als kreativer Input im Sinne einer agilen Entwicklungsmethode berücksichtigt und ist somit Teil der Entwicklung bzw. Auswahl geeigneter Software-Werkzeuge der Kollaborationsumgebung im fortlaufenden Projekt.

## Ausblick

Die Fähigkeit zur befristeten, kollaborativen und digitalen Zusammenarbeit mit unterschiedlichen und zum Teil unbekannten Personen, ist eine zentrale Zukunftsfähigkeit. Hochschulen sind gefordert, darauf ausgerichtete Lehr-Lern-Situationen bereit zu stellen. Dies lässt sich mit dem Wunsch von Studierenden verknüpfen, stärker interdisziplinär und unmittelbar anwendungsbezogen zusammenzuarbeiten und erlaubt es kompetenzorientiertes Lernen adäquater umzusetzen. Im Sinne der wohlbekannten, überarbeiteten Taxonomie (Anderson et al. (2001)) werden somit höhere Stufen adressiert.

Ein Weg zur Umsetzung sind geeignete und angemessen komplexe Aufgabenstellungen, die kollaborativ in Kleingruppen gelöst werden können. Der Beitrag stellt die Hypothese auf, dass dadurch der Grad der (gemeinschaftlichen) Aktivität erhöht und die Hemmschwelle einer intensiven Beschäftigung mit technischen Studieninhalten gesenkt wird. Um diese Hypothesen im Rahmen eines Lehrforschungsprojektes prüfen zu können, werden für zwei Veranstaltungen im Bereich Datenbanksysteme und Data Science konkrete Lehr-Lernkonzepte entwickelt und mit Studierendengruppen umgesetzt.

Technisch wird eine Infrastruktur eingesetzt, die kollaboratives Arbeiten an Fragestellungen ermöglicht. Zur Umsetzung dieser bieten sich aufgrund ihrer Anschaulichkeit, einfachen Bedienbarkeit und vielfältigen Einsatzmöglichkeiten Computational Notebooks an, wie sie in CoCalc, Zeppelin oder Jupyter zur Verfügung gestellt werden.

Sowohl die Entwicklung der Lehr-Lern-Konzepte als auch die Konstruktion und Umsetzung der technischen Plattform sind Bestandteil des Anfang 2021 gestarteten Projektes IoHubHN an der Hochschule Niederrhein. Zur Prüfung der Lehrforschungsfragestellungen werden im Rahmen des Projekts begleitend zu den Lehrveranstaltungen strukturierte Studierendenbefragungen mit vertiefenden Fokusgruppengespräche konzipiert und durchgeführt. Dabei zeigt sich bisher bereits, dass existierende Lehr-Lern-Konzepte auf die untersuchte Fragestellung angepasst werden können und eine technische Umsetzung mit Hilfe von Open-Source-Software grundsätzlich machbar ist.

Es lassen sich mit Bezug auf die eingangs formulierten Fragestellungen nach dem Grad gemeinschaftlicher Aktivität (1) und der Bereitschaft, technische Studieninhalte anzunehmen (2), erste Ergebnisse aus der prototypischen Anwendung und Workshops mit Studierenden erkennen. Studierende stehen dem Konzept und der technischen Umgebung grundsätzlich positiv gegenüber und sind auch bereit, dieses anzuwenden. Prototypisch umgesetzte Lehr-Lernszenarien im Sommersemester 2021 führten in beiden vorgestellten Veranstaltungen zu intensivem Austausch der Studierenden. Einzelne Gruppen nutzen die Plattform auch ohne aktive Betreuung durch Dozierende im Rahmen der Prüfungsvorbereitung. Die aktive Formulierung von Vorschlägen durch Studierende für gewünschte Funktionen lässt die Vermutung zu, dass Studierende in bestimmten Konstellationen einen Mehrwert antizipieren. Die bisherigen Ergebnisse des Projekts zeigen, dass diese Funktionen auch abbildbar sind (z. B. „Nutzer-Cursor“) und genutzt werden.

Nach einer umfänglichen prototypischen Umsetzung werden die Konzepte und Inhalte weiter ausgearbeitet und in größerem Umfang in Lehrveranstaltungen eingesetzt, sodass sich im Rahmen lehrbegleitender Forschung belastbare Aussagen zu Ausmaß und Intensität kollaborativer Arbeit treffen lassen. Ergänzend werden Perspektiven erarbeitet, in welcher Form die Lehr-Lern-Konzepte und die technische Umsetzung auch in Veranstaltungen anderer Studienrichtungen eingesetzt werden können, beispielsweise zur kollaborativen Arbeit bei der Berechnung deskriptiver Kenngrößen oder der Datenexploration in Grundlagenveranstaltungen der Statistik.

## Übersicht referenzierter Software

**Tab. 1:** Übersicht referenzierter Software

Apache Spark	https://spark.apache.org/
Apache Zeppelin	https://zeppelin.apache.org/
Cocalc	https://doc.cocalc.com/
CodeWithMe	https://www.jetbrains.com/code-with-me/
Databricks Notebooks	https://docs.databricks.com/notebooks/index.html
DeepNote	https://deepnote.com/
Docker	https://www.docker.com/
GitLab	https://about.gitlab.com/
Google Colab	https://colab.research.google.com/
Google Docs	https://docs.google.com/
H5P	https://h5p.org/
Jitsi	https://meet.jit.si/
Jupyter	https://jupyter.org/
Kubernetes	https://kubernetes.io/
Moodle	https://moodle.org/

## Footnotes

1 Eine Liste aller im Text genannter Software und der Adressen der zugehörigen Webseiten findet sich im Anschluss an den Text des Artikels.
